# Temporal and spatial variation in reproductive benefits in a partial migrant

**DOI:** 10.1002/ecy.4451

**Published:** 2024-10-25

**Authors:** Stephanie Witczak, Urs G. Kormann, Benedetta Catitti, Patrick Scherler, Valentijn van Bergen, Martin U. Grüebler

**Affiliations:** ^1^ Ecological Research Unit Swiss Ornithological Institute Sempach Switzerland; ^2^ Department of Evolutionary Biology and Environmental Studies University of Zurich Zurich Switzerland; ^3^ Applied Research Unit Swiss Ornithological Institute Sempach Switzerland

**Keywords:** competition, elevational gradient, fledgling number, migration strategy, *Milvus milvus*, red kite, relative reproductive output

## Abstract

In partial migrant systems, where residents and migrants coexist within a population, residents are commonly predicted to gain a reproductive advantage over migrants through priority access to high‐quality territories and an earlier breeding start. Annual variation in reproductive benefits has been suggested to be important for the coexistence of both strategies in a population, as differences in wintering conditions experienced by the two strategies may result in a periodic reproductive advantage for migrants. However, the importance of spatial environmental variation for reproductive output in partially migrant populations remains largely unexplored. We investigated variation in the reproductive output of migrants and residents in a population of Swiss red kites (*Milvus milvus*) both temporally, across and within years, and spatially, along an elevational gradient. We gathered 4 years of reproductive data combined with 183 GPS‐derived full annual cycles from individuals breeding in the Swiss Alpine foothills. At low, but not high, elevations, residents produced more fledglings than migrants. We also found evidence for annual variation in the reproductive advantage of the two strategies. Furthermore, while reproductive output did decline with a later breeding start, there was no difference in the start of breeding between the two migration strategies. The results of this study suggest that differences in reproductive output between migrants and residents in partial migrant populations can vary both due to the use of spatially distinct overwintering grounds and because the strategies are differently affected by spatial variables in the breeding area, such as elevation. The study emphasizes that spatial and temporal variation in reproductive benefits must be considered when predicting how migratory species will respond to future environmental change.

## INTRODUCTION

Reproductive output is one of the key elements contributing to individual fitness and influencing population productivity and dynamics. Different patterns of seasonal migration can be related to reproductive output and, thus, can have a significant influence at both the individual and population levels (Bai et al., 2012; Grist et al., 2017; Marra et al., 1998; Newton, 2008; Norris et al., 2004). In some migratory populations, the propensity to migrate can vary between individuals, resulting in both migrants and year‐round residents coexisting. This is known as partial migration (Lack, 1944; Terrill & Able, 1988), and it occurs in multiple forms in the animal world (Berg et al., 2019; Chapman et al., 2011; Jahn et al., 2012; Menz et al., 2019). Partial migration can occur due to both fixed and changing migration strategies within individuals, arising from genetic variation and phenotypic plasticity (Berg et al., 2019; Lundberg, 1987; Pulido, 2011). There are examples of both latitudinal and altitudinal partial migration, as well as breeding and wintering sympatry among partial migrant systems. Across all systems, a key question is how fitness costs and benefits balance out to facilitate a coexistence of migrants and residents (Berg et al., 2019; Chapman et al., 2011; Jahn et al., 2012; Menz et al., 2019).

Partial migration is commonly believed to be adaptive and shaped by a maximization of fitness in terms of survival and/or reproduction. Seasonal nonbreeding migrants are often expected to gain a survival advantage over residents due to more hospitable overwintering conditions, while residents are expected to gain a reproductive advantage through persistent presence on the breeding grounds (Buchan et al., 2020; Kokko, 2011; Lundberg, 1987). Resident reproductive benefits may include the acquisition of higher quality breeding territories, improved breeding resource‐holding capacity, as well as the ability to initiate breeding earlier than migrants (Aebischer et al., 1996; Ketterson & Nolan, 1976; Kokko, 1999, 2011; Newton, 2008). Most studies on partial migrants still focus largely on factors driving migratory decisions, rather than quantifying the consequences of those decisions (but see Gillanders et al., 2015; Grist et al., 2017; Rolandsen et al., 2017). Moreover, very few studies have examined whether differences in reproductive output between migration strategies vary across environmental gradients (but see Morrissey, 2004). Such studies will help us better understand the occurrence and magnitude of migratory behavioral variation and the success of different migration strategies within and between populations and species.

Reproductive output generally varies in response to changing environmental conditions (Fuller, 2012; Massemin‐Challet et al., 2006; Nägeli et al., 2022) and often exhibits a negative correlation with elevation (Badyaev & Ghalambor, 2001; Grüebler et al., 2010; Morrissey, 2004). Elevational gradients can result in within‐population variation in phenology, competition, and predation (Boyle, 2008; Boyle et al., 2016; Grüebler et al., 2021; Hille & Cooper, 2015), which can, in turn, affect reproduction (Badyaev, 1997; Badyaev & Ghalambor, 2001; Bründl et al., 2020). In temperate systems, where higher elevations are generally colder and, thus, experience harsher winter conditions, residents in high‐elevation territories could experience negative carry‐over effects to the breeding season not experienced by residents with low‐elevation territories. By contrast, such differences across elevation would not be expected for migrants, which do not remain in the breeding area over winter and are, therefore, not exposed to an elevational gradient in winter harshness (Acker et al., 2021; Boyle et al., 2016; Harrison et al., 2011; Norris et al., 2004; Porter et al., 1983).

Existing partial migration theory mainly accounts for temporal, but seldom spatial (e.g., elevational), variation when considering the relative reproductive output (RRO) of migration strategies, that is, the quantitative difference in reproductive output between the two strategies. Furthermore, the underlying mechanisms driving temporal and spatial variation might be different between migration strategies and vary with age in populations where individuals may change migration strategy as they become older (Witczak, Kormann, Schaub, et al., 2024). We suggest three nonexclusive mechanisms that can function individually or in combination to influence spatiotemporal variation in RRO.

### Breeding habitat hypothesis

**FIGURE 1:**
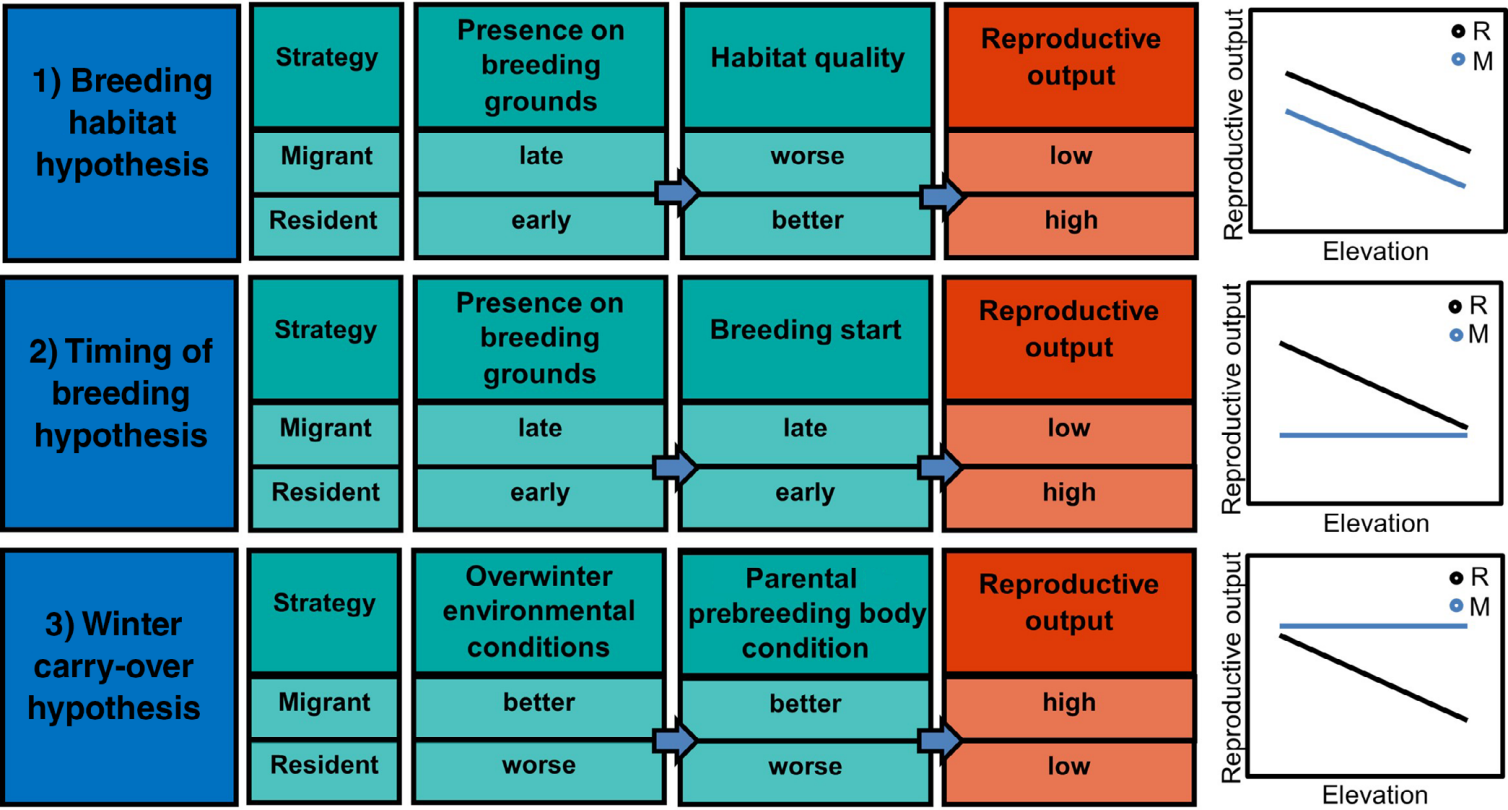
Three hypotheses explaining the expected relative reproductive output between migrants (M) and residents (R). (1) *Breeding habitat hypothesis*: Residents acquire higher quality territories and show higher reproductive output across elevations than migrants. Harsh conditions at high elevations result in lower reproductive output in both strategies. (2) *Timing of breeding hypothesis*: Residents start breeding earlier than migrants and, therefore, show higher reproductive output. This effect disappears at high elevations, where conditions are favorable later. Migrants arrive later and breed at similar times across elevations, exhibiting similar reproductive output. (3) *Winter carry‐over hypothesis*: Harsh winter conditions at high elevations result in lower subsequent reproductive output in residents. Migrants overwinter in milder conditions, independent of nest‐site elevation, and experience higher, elevation‐independent reproductive output.

Having remained in or near their breeding territory over winter, residents have first access to territories come spring (Ketterson & Nolan, 1976, 1983) and benefit from prior residency when defending them (Forstmeier, 2002; Jakobsson, 1988; Kokko, 2011). Consequently, they will acquire higher quality breeding habitats than migrants (ideal despotic distribution; Fretwell & Lucas, 1969; Sergio & Newton, 2003). Therefore, under the breeding habitat hypothesis we expect that (1) residents should exhibit higher reproductive output than migrants across elevations, (2) residents should have a constant reproductive advantage across years, (3) reproductive output declines with increasing elevation in both migration strategies due to colder temperatures and a lower proportion of foraging habitat at higher elevations (Figure 1), and (4) there is an overrepresentation of residents at low elevations.

### Timing of breeding hypothesis

In many species, earlier breeders have higher reproductive output than late breeders (e.g., Møller, 1994; Naef‐Daenzer et al., 2001; Weiser et al., 2018; Wiggins et al., 1994). Furthermore, timing of breeding is linked to nest‐site elevation due to climatic differences affecting spring phenology, which occurs progressively later at higher elevations (Boyle et al., 2016; Hille & Cooper, 2015). In migrants, we expect that the start of breeding will be more limited by the timing of their spring return than by elevation‐related climate in the breeding area (Lok et al., 2017; Martin & Wiebe, 2004; Robson & Barriocanal, 2011). Therefore, under the timing of breeding hypothesis we expect that (1) residents should be able to start breeding earlier at lower elevations and, therefore, exhibit higher reproductive output than residents at higher elevations and higher reproductive output than migrants across all elevations; (2) the timing of breeding in migrants should be determined by arrival time, which is independent of breeding elevation. Because red kites exhibit very high breeding territory fidelity, arrival time is unlikely to influence breeding elevation (e.g., birds do not nest at lower elevations when they return earlier). We, therefore, expect little elevational pattern in the timing of breeding and no elevational gradient in reproductive output (Figure 1). The breeding habitat hypothesis and timing of breeding hypothesis are, therefore, distinct due to their contrasting predictions for the reproductive patterns along an elevational gradient.

### Winter carry‐over hypothesis

In contrast to the above hypotheses, the resident strategy is predicted to have reproductive costs. Poor environmental conditions on the overwintering grounds will affect prebreeding body condition and, thus, reproduction in both migration strategies (Acker et al., 2021; Boyle et al., 2016; Harrison et al., 2011; Norris et al., 2004; Porter et al., 1983). Migrants should face milder conditions in their overwintering areas than are experienced by residents on the breeding grounds, and conditions on the breeding grounds will be milder at low elevations than at high elevations (Martin & Wiebe, 2004). Therefore, under the winter carry‐over hypothesis, we expect that (1) migrants exhibit, on average, higher reproductive output than residents; (2) annual variation in overwintering conditions can lead to annual variation in reproductive output that could favor one migration strategy over the other in different years, dependent on their relative overwintering conditions; and (3) residents at low elevations show higher reproductive output than those at high elevations, while migrants show no elevational pattern in reproductive output because their winter conditions elsewhere are not affected by the elevation of their breeding area, and the winter carry‐over hypothesis does not assume (as the breeding habitat hypothesis does) that breeding habitat quality decreases with elevation (Figure 1).

In this study, we investigated differences in reproductive output between migration strategies. To accomplish this, we used GPS loggers to track red kites (*Milvus milvus*), a nonbreeding partial migrant exhibiting within‐individual plasticity in a migration strategy, breeding along an elevational gradient on the northern slope of the Alps. This study provides insights into the mechanisms underlying the variation in reproductive benefits within migration strategies and how that affects the RRO between strategies. The consideration of both temporal and spatial variation in reproductive output elaborates on a largely unexplored avenue in partial migration research.

## MATERIALS AND METHODS

### Study species and area

**FIGURE 2:**
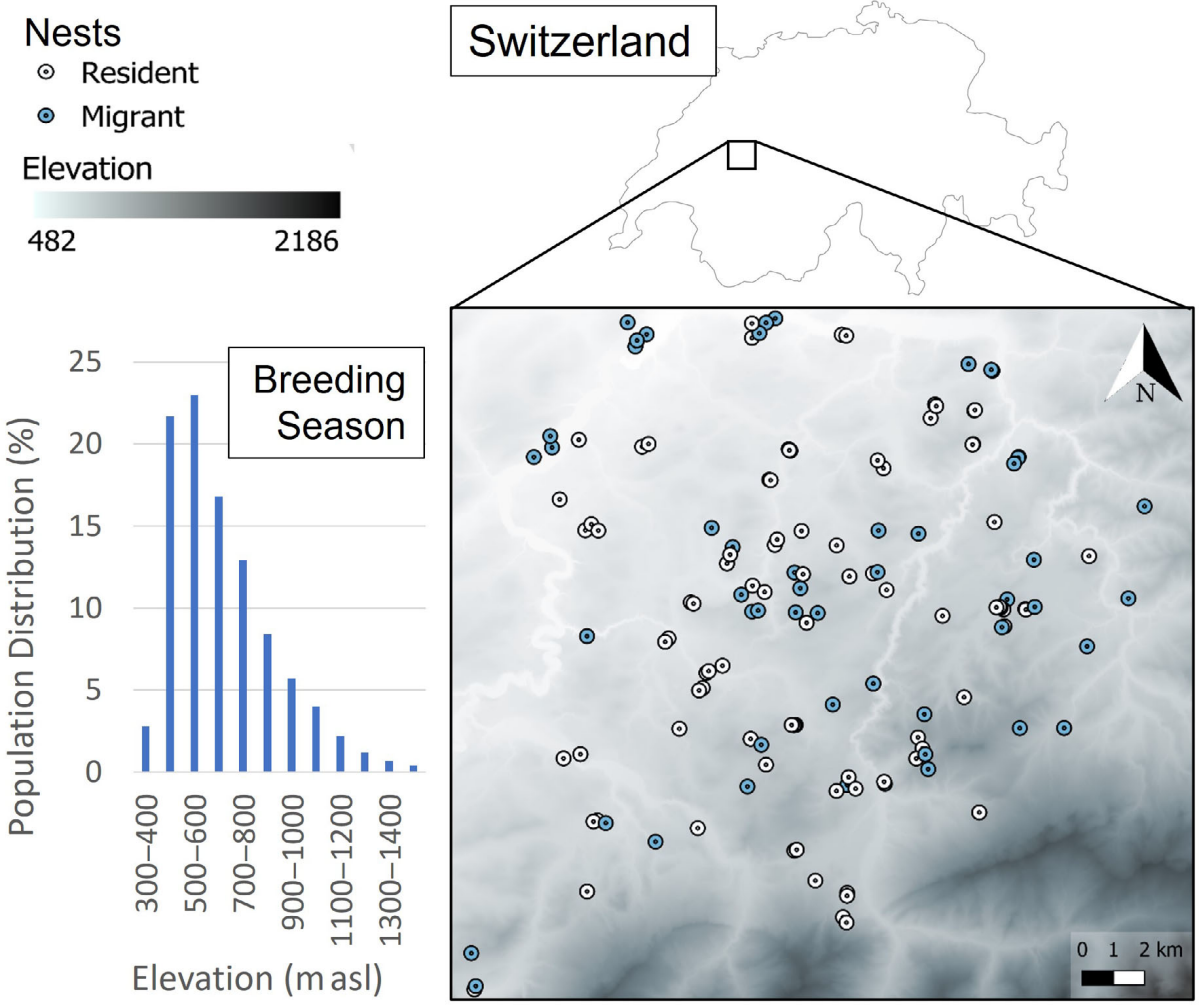
Study area in western Switzerland, spanning 576 km^2^. An elevational gradient of 1704 m (482–2186 m above sea level [asl]) runs along a roughly north–south axis. Nests of residents and migrants are indicated by white and blue points, respectively. Extent: 7.12, 46.69: 7.44, 46.90 EPSG:4326 (elevation map: Federal Office of Topography, swisstopo; Switzerland map: ©Vemaps.com). Switzerland‐wide elevational breeding population distribution (bottom left; Knaus et al., 2018)—note that this histogram is not corrected for the actual area available at different elevations and, therefore, cannot be interpreted as red kites avoiding low‐elevation areas. See Appendix S1: Figure S1 for the elevational distribution of migrants and residents within our sample population.

The red kite is a long‐lived, partially migrant European raptor that occurs from sea level to 2000 m asl with no particular adaptation to montane habitats. In contrast to populations in other areas of Europe, the Swiss population has been increasing in number for approximately 40 years, expanding upward in elevation toward the Alps (Aebischer & Scherler, 2021). The Swiss population is currently estimated to contain around 3500 breeding pairs (Knaus et al., 2018). Swiss migrants overwinter in France, Spain, and Portugal, while residents have become increasingly abundant since the 1950s, with numbers at Swiss winter roosts climbing to 3000–5000 in 2020 (Aebischer & Scherler, 2021). Previous work on this population showed that nearly all juveniles migrate in their first winter (97.4%), but this proportion decreases significantly with age and reaches a stable proportion when birds are approximately 10 years old (35.9% of adults still migrate; Witczak, Kormann, Schaub, et al., 2024). Furthermore, once resident, switching back to migrant rarely occurs. Our study area is situated in western Switzerland, primarily within the Sense District of Canton Freiburg, covering c. 576 km^2^, and ranging from 482 to 2186 m asl (Figure 2). Elevation increases toward the south, paralleled by an increase in pastureland and forest cover, and a decrease in human occupancy (for details, see Scherler, Witczak, et al., 2023 and references therein). The study area is part of a highly productive region for red kites (1.62 ± 0.65 fledglings per successful brood, 2015–2020; Scherler, van Bergen, et al., 2023), with an average of 32 breeding pairs per 100 km^2^ (Knaus et al., 2018). In our population, recruitment into the breeding population was observed no earlier than the third calendar year, with most individuals recruiting later. Red kites typically lay two to three eggs and although chicks hatch 2–3 days apart and the last‐hatched chicks often have the lowest survival prospects (Catitti et al., 2022; Nägeli et al., 2022), there is no obvious sacrificial chick and red kites can raise up to three fledglings (Scherler, van Bergen, et al., 2023). Brood loss is most often due to adverse environmental conditions, with predation playing only a small role (Nägeli et al., 2022; Scherler, van Bergen, et al., 2023).

### Timing of breeding

We performed behavioral surveys from March to June to identify territorial couples. We located nests and monitored them until incubation. At some, we mounted camera traps (Reconyx HC500 HyperFire; RECONYX, Inc.) or webcams (Microsoft LifeCam Cinema, 5Mpx, Microsoft) to determine the exact timing of breeding within the season. We defined timing of breeding as the start of incubation, derived from one of three sources: nest cameras (*n* = 12 nests), field observations (*n* = 35), or the back‐dated initiation date inferred from the size of nestlings and their typical growth rate (*n* = 134). We used the growth rate of the 8th primary feather established through 139 measurements of 49 known‐age chicks (Nägeli et al., 2022) to estimate hatching time, and inferred the start of incubation by subtracting 32 days from hatching as the mean incubation length (Scherler, van Bergen, et al., 2023; details in Appendix S1: Section S1).

### Capture and logger‐fitting

Once nestlings were c. 10 days old, we used a Dho‐Gaza net in combination with a live decoy predator (Eurasian eagle owl, *Bubo bubo*) to capture the parents (details in Witczak, 2023). A tree climber visited nests prior to fledging when nestlings were c. 35–45 days old. We avoided climbing when nestlings were older than c. 45 days to prevent premature fledging. We measured wing length, P8, tarsus length (in millimeters), and weight (in grams) in all birds, and took a blood sample for molecular sexing (for details, see Fridolfsson & Ellegren, 1999; Nägeli et al., 2022). We equipped birds with solar‐powered GPS‐GSM‐UHF loggers (SKUA/CREX, Ecotone Telemetry, Gdynia, Poland, or GsmRadioTag‐M9, Milsar, Cluj Napoca, Romania), using a backpack‐style diagonal‐loop harness (Kenward, 1985; Meyburg & Fuller, 2007). Loggers and harness weighed maximally 3.2% of the body weight of the lightest adult red kite; see Scherler, Witczak, et al. (2023) for details. GPS data collected by the loggers (sampling regime: >1 location/h, 5:00–21:00 h) were transmitted using GSM, 2G, or 3G networks, and these data were subsequently saved on and managed through Movebank (Wikelski et al., 2024). Ringing and tagging was permitted by the Canton of Fribourg Amt für Lebensmittelsicherheit und Veterinärwesen (Permit No. 2015_13_FR and 2017_29_FR) and the Swiss Federal Office for the Environment.

### Reproductive output

We defined fledgling number as the number of nestlings still alive at the end of the nestling period (after 35–45 days), as counted by the tree climber at the final climb before fledging (range = 0–3 fledglings). RRO was calculated as the difference in fledgling number between residents and migrants such that positive numbers indicate a resident advantage.

### Migration strategy

We assigned individuals to one of two migration strategies per year: resident, where the bird remained in or near their breeding territory year‐round; or migrant, where the bird flew to southwestern Europe in winter. We used a rule‐based approach to determine the migration strategy and spring arrival dates of migrants (Julian date 1 = January 1 of previous year) by chronologically plotting the mean daily displacement of GPS locations during the fall and winter from the most recent nest site. In the case of first‐time breeders, we used the nest of origin. See Scherler (2020) and Witczak, Kormann, Schaub, et al. (2024) for details.

### Statistical analyses

We were interested in the effect of migration strategy on reproductive output, and plausible mechanisms leading to differences between strategies. We, therefore, first modeled the effect of migration strategy on the number of fledglings (= reproductive output). We then explored the effect of migration strategy on two additional parameters related to reproduction that could provide a plausible mechanism for differences in reproductive output. These two additional variables were nest‐site elevation (a continuous measure of meters above sea level [asl]) and timing of breeding (a continuous measure of Julian date, where Julian date 1 = January 1 of the current year). We modeled timing of breeding using the whole dataset, as well as a dataset consisting of only migrants to investigate the effect of spring arrival date.

We further included nest‐site elevation and timing of breeding as predictor variables when modeling reproductive output. We included year as a categorical predictor variable (2017–2020) in all models to account for potential annual variation in environmental conditions. We also included parental body size, estimated using tarsus length (which reaches final length before fledging; Viñuela & Ferrer, 1997), as a continuous predictor variable in all models in order to control for a potential effect of size on reproduction (Choudhury et al., 1996; Sergio et al., 2007a, 2007b). To account for observer differences and a marginal sexual size dimorphism in red kites, we did not use the raw measured values of tarsus length, but first subtracted the mean tarsus length measured by each observer from the size observations recorded by them, then subtracted the mean centered tarsus length of each sex from the centered size observations depending on an individual's sex (resulting in females: mean = 0.07 ± 2.52, range = −5.28, 4.25; males: mean = −0.06 ± 3.39, range = −7.56, 10.65; see Aebischer & Scherler, 2021). Finally, we controlled for the effect of breeding experience by including a binary variable (first‐time or experienced breeder) in all models, as first‐time breeders have been shown to differ from experienced breeders in reproductive timing and output (Blas et al., 2009; Espie et al., 2000; Gauthier, 1989; Sydeman et al., 1991). First‐time breeder status was determined only for individuals followed from fledging via GPS. We scaled all continuous explanatory variables before modeling by subtracting the mean of the variable and dividing by its SD.

We analyzed factors affecting the nest‐site elevation and timing of breeding using linear mixed effect models with a Gaussian distribution (“lmer” in package lme4: Bates et al., 2015). We analyzed factors affecting fledgling number using a generalized linear mixed effect model with a Conway‑Maxwell Poisson distribution to correct for zero‐inflation (proportion of nests with zero fledglings = 0.28) and under‐dispersion likely caused by the small range of response values (“glmmTMB” in package glmmTMB: Brooks et al., 2017). Where both parents of a nest were tagged, we randomly removed one parent per year to avoid pseudo‐replication. Partners typically use the same migration strategy. We implemented either couple‐specific or nest‐specific random intercepts to account for the hierarchical structure in the data (detailed in Tables 1 and 2). We first included the explanatory variables as main effects in the models as outlined above. When modeling nest‐site elevation, we excluded timing of breeding as nest‐site decisions occur prior to laying.

**TABLE 1:** Estimates and 95% credible intervals (CrI) of the linear mixed effect models investigating nest‐site elevation and timing of breeding in all birds and migrants only.

Model	Nest‐site elevation			Timing of breeding					
				All birds			Migrants only		
	β	95% CrI		β	95% CrI		β	95% CrI	
Intercept	736.33	701.62	770.70	96.59	91.69	101.32	97.45	93.76	101.19
Strategy (migrant)	18.55	−8.06	45.90	0.70	−5.60	6.99	…	…	…
Return date	…	…	…	…	…	…	**2.95**	**1.04**	**4.85**
Body size	**−17.64**	**−30.42**	**−4.87**	**−1.63**	**−3.13**	**−0.11**	−1.72	−4.51	1.03
Year (2018)	6.89	−14.15	28.05	3.96	−1.42	9.41	0.66	−3.98	5.43
Year (2019)	16.51	−4.29	37.08	−1.89	−7.18	3.45	0.27	−4.53	5.22
Year (2020)	11.35	−9.36	31.93	−0.32	−5.61	5.02	−3.79	−8.88	1.42
Experience (first‐time breeder)	21.97	−3.91	47.82	**8.15**	**3.93**	**12.28**	**5.76**	**0.74**	**10.89**
Elevation	…	…	…	1.43	−0.09	3.01	1.06	−1.16	3.22
Migrant × 2018	8.06	−22.70	39.31	−3.38	−11.12	4.32	…	…	…
Migrant × 2019	−12.02	−46.21	21.16	1.71	−6.01	9.28	…	…	…
Migrant × 2020	−4.47	−40.56	31.40	−3.17	−10.90	4.65	…	…	…

*Note*: Random effects: Nest‐site elevation model: SD_coupleID_ = 123.10; timing of breeding model (all birds): SD_nestID:coupleID_ = 5.16; timing of breeding model (all birds): SD_coupleID_ = 2.36; timing of breeding model (migrants only): SD_nestID:coupleID_ = 5.98; timing of breeding model (migrants only): SD_coupleID_ = 2.33. Conditional *R* ^2^: Nest‐site elevation model: 0.96; timing of breeding model (all birds): 0.59; timing of breeding model (migrants only): 0.84. CrIs that do not overlap 0 are denoted in boldface.

**TABLE 2:** Estimates and 95% credible intervals (CrI) of the generalized linear mixed effect model investigating fledgling number.

Variable	β	95% CrI	
Intercept	−0.17	−0.65	0.32
Strategy (migrant)	**0.63**	**0.07**	**1.20**
Body size	0.10	−0.03	0.23
Year (2018)	**0.55**	**0.02**	**1.08**
Year (2019)	0.20	−0.33	0.73
Year (2020)	0.35	−0.15	0.88
Experience (first‐time breeder)	**−1.04**	**−1.76**	**−0.30**
Elevation	0.02	−0.16	0.19
Timing of breeding	**−0.18**	**−0.31**	**−0.06**
Migrant × 2018	−0.62	−1.29	0.03
Migrant × 2019	**−0.86**	**−1.64**	**−0.09**
Migrant × 2020	**−0.85**	**−1.61**	**−0.11**
Migrant × elevation	**0.24**	**0.02**	**0.45**

*Note*: Random effect: SD_coupleID_ = 0.18; conditional (pseudo) *R* ^2^ = 0.43; dispersion parameter = 0.39. CrIs that do not overlap 0 are denoted in boldface.

To test whether the benefit of migration varies across elevations or among years (as predicted by the breeding habitat, timing of breeding and winter carry‐over hypotheses; see Figure 1), we included interactions between migration strategy and elevation, and migration strategy and year in all models, and kept them in the model investigating the factors affecting reproductive output as they tested our hypotheses. However, because unimportant interactions may impede the interpretation of main effects (Engqvist, 2005), we eliminated unimportant interactions that increased the Akaike information criterion for small samples but retained all main effects in the other models. When modeling timing of breeding in migrants, we replaced the variable “migration strategy” with “spring arrival date,” as only migrants were included in this analysis, and removed all associated interaction terms. In all tables and visualizations of results, unless otherwise indicated, the reference categorical variables were migration strategy = resident, year = 2017, and breeding experience = experienced breeder. Numeric variables not plotted were held at their mean.

Spatial residual maps (“bubble” in package sp.: Bivand et al., 2013; Pebesma & Bivand, 2005) indicated no spatial autocorrelation in the models. We calculated 95% credible intervals (denoted: CrI or using []) for parameter estimates, model predictions, and derived statistics by drawing 10,000 random samples from the posterior distribution of the model parameters (“sim” in package arm: Gelman & Yu‐Sung, 2020; Korner‐Nievergelt et al., 2015). We carried out all data processing and analyses in RStudio version 1.3.959 (RStudio Team, 2020), using R version 4.0.2 (R Core Team, 2020).

## RESULTS

Between 2016 and 2018, we equipped 78 breeding red kites with GPS loggers. Additionally, 20 individuals that were fitted with loggers as juveniles between 2015 and 2017 were recruited into the local breeding population and were included in the sample. We removed 12 individuals that either died, lost loggers, had missing parameters, or whose loggers had failed, and it is unknown what migratory strategy these individuals might have pursued. A further five individuals were removed to avoid pseudo‐replication within pairs. This left us with a final sample of 183 full annual cycles (overwintering and subsequent reproduction) across 81 individuals (mean ± SD = 2.26 ± 1.05 annual cycles/individual, range = 1, 4). Of the 183 annual cycles, 64% (*n* = 118) were resident and 36% migrant (*n* = 65). Within the migrant‐only dataset, 65 annual cycles from 43 individuals were available (mean ± SD = 1.51 ± 0.70 annual cycles/individual, range = 1, 3, Appendix S1: Table S1).

### Nest‐site elevation

Migrants and residents nested at similar elevations (migrants: mean ± SD = 765 ± 147 m asl, range = 503, 1050; residents: mean ± SD = 748 ± 123 m asl, range = 519, 1087). Model contrasts presented support for slightly higher nest‐site elevations in migrants in 2018, although the effect of the migration strategy × year interaction was not different from 0 (Table 1; contrasts: β_2017_ = 18.55 [−8.06, 45.90] m difference in migrants versus residents; β_2018_ = 26.61 [5.67, 48.12]; β_2019_ = 6.53 [−16.57, 30.03]; β_2020_ = 14.08 [−12.23, 40.88]). Furthermore, nest‐site elevation was influenced by body size, with smaller individuals nesting at higher elevations (Table 1).

### Timing of breeding

Migrants and residents started breeding at similar times (migrants: Julian date mean ± SD = 98.08 ± 8.25, range = 82, 125; residents: Julian date mean ± SD = 96.53 ± 9.10, range = 77, 135). Contrasts indicated no differences in the timing of breeding between migration strategies in any year (Table 1; contrasts: β_2017_ = 0.70 [−5.60, 6.99] days difference in migrants versus residents; β_2018_ = −2.69 [−7.43, 2.02]; β_2019_ = 2.40 [−2.18, 6.91]; β_2020_ = −2.48 [−7.24, 2.24]). First‐time breeders started breeding 8.15 days [3.93, 12.28] later than experienced breeders and small individuals started breeding later than large individuals (Table 1). We found no difference in the effect of elevation on the timing of breeding between migration strategies, although there was a tendency for breeding to start 1.09 days [−0.06, 2.28] later per 100 m elevational increase. When modeling migrants only, spring arrival date was found to affect timing of breeding (Table 1), with birds starting incubation 1.81 days [0.64, 2.98] later for every 10 days later returned.

### Fledgling number

**FIGURE 3:**
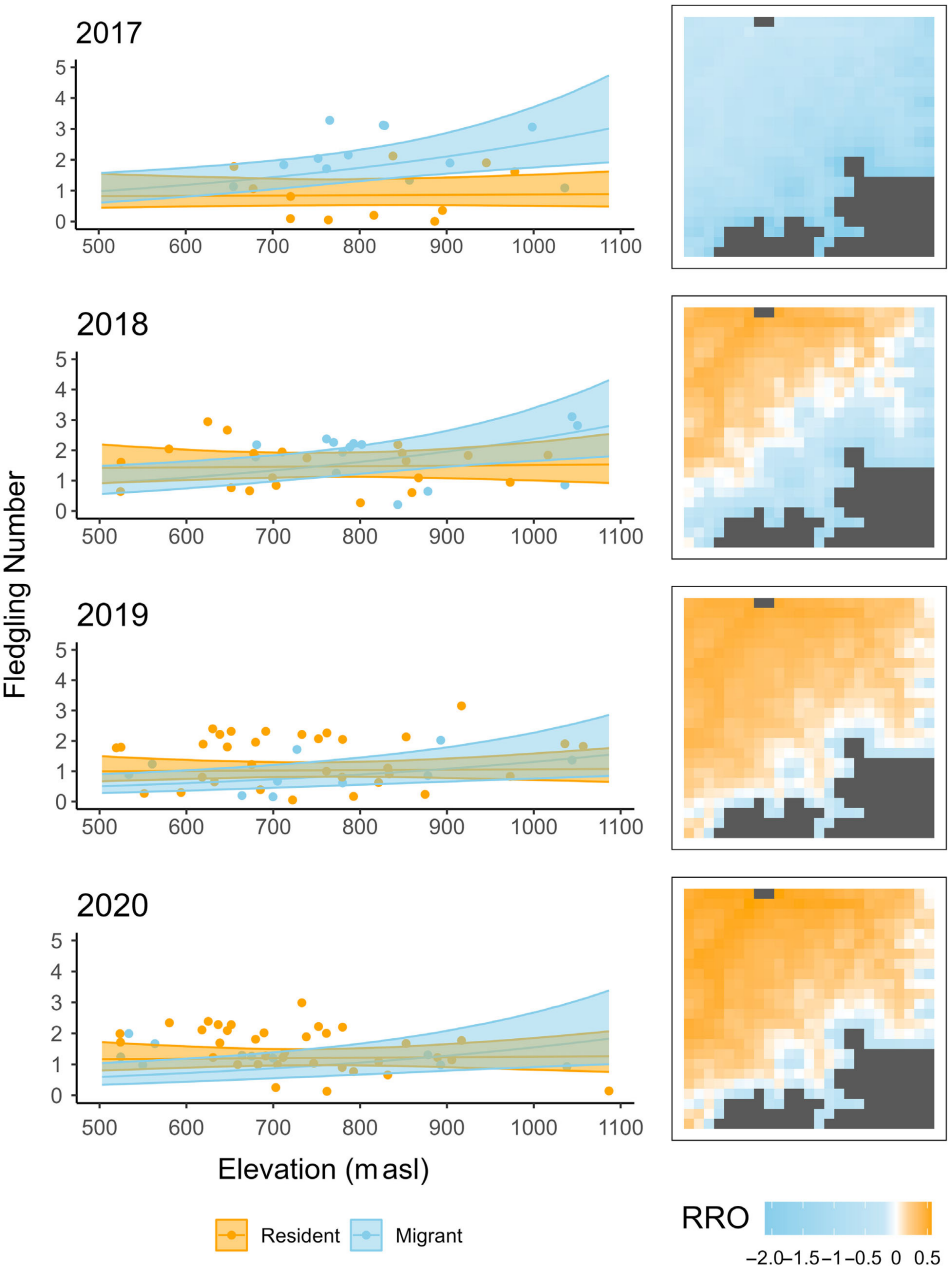
Interactive effect of migration strategy and nest‐site elevation, and migration strategy and year on fledgling number, including 95% credible intervals (CrI), with raw data in the background (left row). Elevational effect on the relative reproductive output (RRO), where RRO >0 indicates a resident advantage and RRO <0 indicates a migrant advantage, plotted across the study area (right row).

**FIGURE 4:**
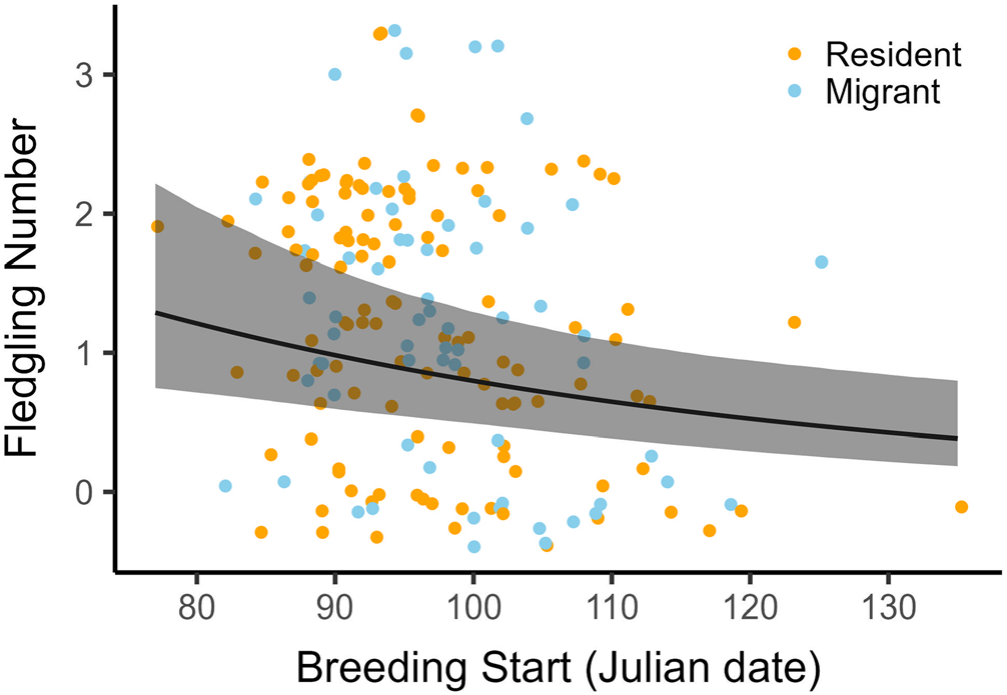
Effect of timing of breeding on fledgling number, including 95% credible intervals (CrI). Julian date: 77 = March 18, Julian date: 134 = May 14. Raw data in the background.

Fledgling number was affected by timing of breeding and breeding experience, and by the interactions between migration strategy and year, and between migration strategy and elevation (Table 2). In 2017, migrants produced 0.63 [0.07, 1.20] more fledglings than residents. In the other years, fledgling number showed no statistical difference between strategies (Table 2, Figure 3; contrasts: β_2018_ = 0.01 [−0.38, 0.41]; β_2019_ = −0.23 [−0.75, 0.29]; β_2020_ = −0.22 [−0.71, 0.26]). Fledgling number of migrants increased with elevation (β = 0.24 [0.02, 0.45]) such that for every 100 m elevation increase migrants produced 0.21 [0.09, 0.27] more fledglings. In contrast, no elevational effect was present in residents (Table 2, Figure 3). The number of fledglings decreased with a later timing of breeding (Table 2, Figure 4), with 0.91 [−1.78, −0.30] fewer fledglings in broods initiated mid‐May compared to mid‐March, while first‐time breeders produced 1.04 [−1.76, −0.30] fewer fledglings than experienced breeders (Table 2).

## DISCUSSION

By combining GPS tracking with multi‐year reproductive surveys, this study elaborates on a largely unexplored avenue in partial migration research: the mechanisms underlying spatiotemporal variation in the reproductive benefits of migration strategies. We found that the RRO of migrants and residents differed considerably in space and time. First, our results showed an increase in reproductive output with elevation in migrants, but not residents. This resulted in migrants showing lower reproductive output than residents at low elevations, but not at high elevations. Even though this elevational pattern was clear, it was contrary to our hypotheses. Second, the results indicated annual variation in the RRO between migrants and residents. This pattern suggests carry‐over effects due to annually variable overwintering conditions, in line with the winter carry‐over hypothesis. Finally, although earlier broods produced more fledglings and timing of breeding in migrants was influenced by spring arrival date, migrants and residents did not differ in the timing of breeding, indicating that residents gained no timing‐related benefits. Therefore, we found no support for the timing of breeding hypothesis. Ultimately, this study provides support for a dynamic landscape of RRO between the migration strategies that goes beyond the separate hypotheses used here to investigate reproductive benefits (differences in fledgling numbers) in partial migration.

Besides migration strategy, many long‐lived animals generally improve their reproductive output with increasing age up to a certain point (Blas et al., 2009; Martin, 1995; Murgatroyd et al., 2018), but the cause of that improvement is often not well understood (Forslund & Pärt, 1995). In our study system, birds increasingly become resident as they mature (Witczak, Kormann, Schaub, et al., 2024), and our analysis of reproductive consequences of migration strategy can, therefore, to some extent be confounded by a decreasing probability to migrate with age. However, although we cannot conclusively demonstrate whether the ultimate causal mechanism for increased reproductive output is increasing residence or age, we argue that a single underlying process might explain both patterns. As birds age, they may be able to better cope with both, more adverse weather conditions in winter, allowing them to reside in temperate breeding areas year‐round and potentially improve survival (Witczak, Kormann, Schaub, et al., 2024), and with temporary food shortages during breeding, thus increasing reproductive output (Weimerskirch, 2018). Thus, rather than considering it as a confounding variable, we suggest that increasing reproductive output with increasing age or residence is a phenomenon of the same underlying process of becoming more experienced with age. Moreover, there was no indication that birds tagged as adults increased their reproductive output during the study period.

### Elevational variation in reproductive benefits

In contrast to our expectations, reproductive output did not decrease with increasing elevation in either migration strategy, nor were residents consistently more successful than migrants across elevation. Instead, we found a positive effect of elevation on reproductive output in migrants, while resident output was not associated with elevation. This resulted in a reproductive advantage for residents at low, but not high, elevations. While an elevational increase in habitat quality would explain the increase in reproductive output of migrants, we would expect the same pattern for residents, which we do not find. We would also expect frequent breeding dispersal to higher elevations were habitat quality higher there (Forero et al., 1999; Grüebler et al., 2021; Newton, 2001), but breeding dispersal is rare in our study population (Scherler, van Bergen, et al., 2023).

A possible mechanism resulting in the observed reduction in migrant reproductive output at low elevations might be disturbance by subadult floaters. In our study population, subadult floaters prefer low elevations (Orgeret et al., 2023; Scherler, 2020) and may use winter roosts in the breeding area to gain first access to opening gaps in the territorial system (Mindt, 2022). Thus, migrants with territories at low elevations may have larger energetic or temporal costs associated with re‐establishing territories upon return than migrants at higher elevations, where competition with recruiting resident subadults is not as high. Residents, who maintain their territories year‐round, are less affected by subadult densities at lower elevations and suffer no reduction in reproductive output. Thus, the high density of resident subadults and competitive interactions with territorial birds may reduce reproductive output in migrants, reinforcing resident benefits at lower elevations (Bretagnolle et al., 2008; Chambert et al., 2020).

Even though residents experienced a reproductive advantage only at lower elevations in our population, the majority of Switzerland's red kite breeding distribution occurs within that elevation range (<700 m; Figure 2; Knaus et al., 2018). Thus, a large proportion of the population may benefit from a reproductive advantage as residents. If this is the case, the resident strategy adds considerably more to the productivity of the Swiss population than the migrant strategy. Indeed, aside from years with the strongest disadvantage for residents, residents at the lowest elevations almost always had an advantage (Figure 3), and due to high breeding densities at low elevations in Switzerland, this would affect a considerable part of the population. This potentially large‐scale reproductive advantage for residents suggests that the occurrence of the resident strategy will increase in the future, as already observed for some decades (Aebischer & Scherler, 2021; Knaus et al., 2018).

### Seasonal variation in reproductive benefits

The onset of breeding was delayed in first‐time breeders and in smaller individuals, with some evidence of delayed breeding at higher elevations (Scherler, Witczak, et al., 2023). The effects of breeding experience and elevation on timing of breeding are well‐documented in other species (e.g., elevation: Boyle et al., 2016; Bründl et al., 2020; experience: Espie et al., 2000; Gauthier, 1989; Sydeman et al., 1991), while size‐related effects may be due to differences in competitiveness, delaying the start of smaller subordinates (e.g., Langston et al., 1990; Sergio et al., 2007a). We found no size difference between first‐time and experienced breeders in our dataset and, therefore, consider it unlikely that the lower competitiveness of first‐time breeders is due to smaller size.

Later broods yielded fewer fledglings in both migrants and residents; however, in contrast to earlier studies (Gillis et al., 2008; Grist et al., 2017; Massemin‐Challet et al., 2006; Morrissey, 2004), timing of breeding was found to be similar between migration strategies, lending no support to the timing mechanism proposed by the timing of breeding hypothesis. This result can be largely explained by spring arrival dates. The mean arrival date across the four study years was 53.37 ± 14.23 days earlier than the mean start of breeding such that even individuals returning late relative to their breeding onset had 1 month for breeding preparation (minimum = 29 days). A similar result was found in partially migrant lesser kestrels (*Falco naumanni*; Buchan et al., 2021). Therefore, the timing of breeding in both migrants and residents was more likely determined by local conditions on the breeding grounds such as weather and food availability (Martin & Wiebe, 2004; Ockendon et al., 2013; Svensson & Nilsson, 1995).

Our results show that the assumption that residents gain an advantage in the timing of breeding by maintaining a territory year‐round (e.g., Bai et al., 2012) does not apply in our study system. The high degree of territory and partner fidelity observed in our population suggests that territory retention rate is not lower in migrants than in residents, and we propose that both strategies benefit from an inter‐annual prior residency effect (Forstmeier, 2002; Kokko, 2011; Kokko et al., 2006). This would give experienced breeders an advantage over usurpers when reclaiming and defending their previous‐year's territory. Nevertheless, migrants may have additional (e.g., energetic) reproductive costs associated with territory retention in areas with high subadult density. Furthermore, timing‐related disadvantages of territory establishment may be present in migrant first‐time breeders, where inter‐annual prior residency plays no role. This suggests that an earlier age of first breeding enhancing lifetime reproductive output might be an important benefit for residents and warrants further study.

### Annual variation in reproductive benefits

In 2017, migrants produced more fledglings than residents, while residents tended to produce more fledglings than migrants in 2019 and 2020. A reproductive advantage for migrants in 1 year suggests a potential role of carry‐over effects from wintering conditions in some years, which could either increase the reproductive output of migrants or decrease the reproductive output of residents if they had to endure poor conditions. Poor weather or food conditions during winter can reduce the prebreeding body condition of individuals and, thus, reproductive output (Acker et al., 2021; Boyle et al., 2016; Harrison et al., 2011; Norris et al., 2004; Porter et al., 1983). In 2017, Switzerland recorded one of the coldest Januarys in 30 years (MeteoSwiss, 2017), likely contributing to the reduced reproductive output of residents, and the Iberian Peninsula showed warmer winter temperatures than average (https://climate.copernicus.eu/climate-2017-european-temperature). We acknowledge that this year also had the lowest sample size in our study, which may affect our confidence in this conclusion. However, such annual variation in environmental conditions affecting reproductive benefits, known also from other species (Bai et al., 2012; Buchan et al., 2021; Hegemann et al., 2015), suggests that long‐term reproductive benefits for one strategy depend on the frequency of adverse wintering conditions on the breeding grounds. The small sample of years in our study prevented the quantification of a predicted decrease in reproductive output in residents after poor overwintering conditions. Therefore, large long‐term studies are needed to investigate spatiotemporal variation in carry‐over effects of overwintering conditions and their effects on reproductive benefits for different migratory strategies.

## CONCLUSIONS

Our results provide clear evidence that the RRO of the migration strategies varies in time and space. Annual variation and spatial gradients in environmental factors allow for a periodic advantage and the occurrence of spatial strongholds in the disadvantaged strategy. The significance of variation in the RRO depends on what proportion of a population is affected over large spatial and long temporal scales. By investigating spatial variation in reproductive benefits, our study contributes to the increasing body of literature that documents how climate change may affect the occurrence of polymorphisms in wild animals (e.g., Karell et al., 2011; Mills et al., 2018), as well as the future of migratory species, in general (e.g., Both et al., 2010; Van Doren, 2022). Winters on temperate breeding grounds are likely to become milder due to climate change (Seneviratne et al., 2021), suggesting reproductive advantages of the migrant strategy may further decrease, with spatial strongholds occurring only in areas of high elevation. Furthermore, despite traditional expectations regarding the advantages of migration strategies, survival benefits have been shown to occur more often than reproductive benefits in residents across taxa (Buchan et al., 2020). In red kites, migrants are being exposed to increasing incidences of human‐related mortality (e.g., poisoning, poaching, collisions; Mattsson et al., 2022), which could contribute to the rapid spread of the resident strategy in this species, also observed in many other temperate zone species (Buchan et al., 2020; Meller et al., 2016; Warkentin et al., 1990). Ultimately, quantifying the spatiotemporal variation in the fitness parameters of migrants and residents is crucial to understanding the mechanisms maintaining partial migration, as well as predicting migratory behavior under future environmental scenarios.

## AUTHOR CONTRIBUTIONS

Stephanie Witczak, Martin U. Grüebler, and Urs G. Kormann conceived the study. Stephanie Witczak, Patrick Scherler, Benedetta Catitti, and Valentijn van Bergen collected the data. Stephanie Witczak analyzed the data with critical conceptual and analytical input from Martin U. Grüebler and Urs G. Kormann. Stephanie Witczak drafted the manuscript with critical input from Martin U. Grüebler and Urs G. Kormann. All authors contributed to the drafts and gave final approval for publication.

## CONFLICT OF INTEREST STATEMENT

The authors declare no conflicts of interest.

## Supporting information


Appendix S1.

## Data Availability

Data and code (Witczak, Kormann, Catitti, et al., 2024) are available in Zenodo at http://doi.org/10.5281/zenodo.13358390.
