# Supplementary material for: Temporal and spatial variation in reproductive benefits in a partial migrant
Source: Ecology. 2024 Oct 25;105(12):e4451. doi: 10.1002/ecy.4451 (PMC11610666; doi:10.1002/ecy.4451)
Supplement: Supplementary file 1 — Appendix S1. [file ECY-105-e4451-s001.pdf]

Journal: Ecology

Title: Temporal and spatial variation in reproductive benefits in a partial migrant

Author names: Stephanie Witczak, Urs G. Kormann, Benedetta Catitti, Patrick Scherler, Valentijn van Bergen, Martin U. Grüebler

Appendix S1

Section S1 Determination of incubation dates
Table S1 Sample sizes of red kites
Figure S1 Proportion of migrants and residents in relation to elevation

Section S1 Determination of incubation dates

By nest-camera ($n_{residents}$ *= 9;* $n_{migrants}$ *= 3):* Either incubation start could be extracted from images, or it was calculated by subtracting the mean duration of incubation within this population (mean = 31.8, SD = 1.2 days; Scherler et al. 2023) from the hatching date of the oldest chick visible.

By nestling measurement ($n_{residents}$ *= 87;* $n_{migrants}$ *= 47):* Hatching date of the oldest chick was inferred from the size of the 8th primary feather and an age estimate of that feather derived from a growth curve based on 139 measurements of 49 chicks of known hatching date in this population (Nägeli, 2019; Nägeli et al., 2022). The mean incubation duration (mean = 31.8, SD = 1.2 days; Scherler et al. 2023) was subtracted from the inferred hatching date to calculate incubation start.

By field observation ($n_{residents}$ *= 21;* $n_{migrants}$ *= 14):* Start of incubation or hatching date was calculated as the midpoint between the last observation without, and first observation with incubation, or without and with nestlings, respectively (see Scherler et al. 2023). Mean incubation duration was subtracted from hatching date to infer incubation start. In cases where the female parent was equipped with a GPS device, and the incubation start date was uncertain, we visually examined the GPS data and adjusted the estimates to improve their accuracy based on her movement patterns ($n_{residents}$ = 1; $n_{migrants}$ = 1). In 16 cases, we had too little information to yield a reliable estimate, and these were excluded from further analyses. Julian dates were used for all analyses.

References

Nägeli, M. 2019. *The quantity and quality of nestlings : how food supplementation and environmental factors influence reproductive traits of red kites*. Master Thesis. University of Zurich.

Nägeli, M., P. Scherler, S. Witczak, B. Catitti, A. Aebischer, V. van Bergen, U. Kormann, and M.U. Grüebler. 2022. "Weather and food availability additively affect reproductive output in

an expanding raptor population.” *Oecologia* 198 (1): 125–138. https://doi.org/10.1007/s00442-021-05076-6.

Scherler, P., V. v. Bergen, B. Catitti, U. Kormann, S. Witczak, M. Andereggen, J. S. Herzog, A. Aebischer, N. Roth, and M. U. Grüebler. 2023. Brutbiologie des Rotmilans *Milvus milvus* in den Westschweizer Voralpen. Ornithologischer Beobachter 120: 276–292.

Table S1 Sample sizes of red kites with available information on migration strategy and reproductive output from central Switzerland between 2017-2020. ‘Young’ and ‘old’ reflect birds that breed for the first time or have bred before, respectively.

Year	Strategy	Old females	Old males	Young females	Young males
2017	migrant	8	6	1	0
2017	resident	4	7	0	0
2018	migrant	10	6	1	1
2018	resident	10	12	1	1
2019	migrant	5	7	3	2
2019	resident	19	20	0	1
2020	migrant	5	6	1	3
2020	resident	21	19	3	0

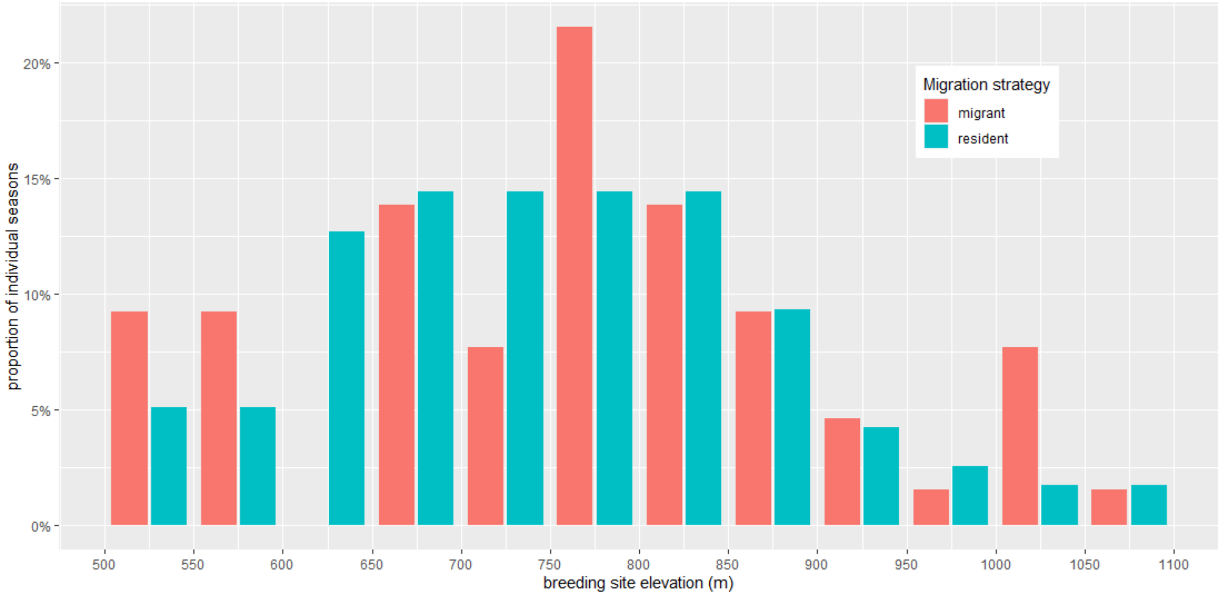

Figure S1 Proportion of individual breeding seasons of migrants and residents in 50 m elevation bins showing the similar distribution of migrants and residents across the elevational gradient of the study area in Switzerland.
